# Effects of transcranial direct current stimulation over frontal, parietal and cerebellar cortex for cognitive function during fasting in healthy adults

**DOI:** 10.1016/j.ibror.2020.03.002

**Published:** 2020-04-25

**Authors:** Fahad Alsultan, Malak Alaboudi, Abdullah Almousa, Reema Alajaji, Shahid Bashir

**Affiliations:** a Department of Medicine, King Saud Medical City, Riyadh, Saudi Arabia; b Prince Sultan Military Medical City, Riyadh, Saudi Arabia; c Department of radiology, King Fahad Medical City, Riyadh, Saudi Arabia; d Neuroscience Center, King Fahad Specialist Hospital, Dammam, Saudi Arabia

**Keywords:** Ramadan fasting, Cognitive functions, Transcranial direct current stimulation, Attention

## Abstract

**Background:**

Transcranial direct current stimulation (tDCS) is a neuromodulation tool used to modify the cognitive function in subjects. There is a paucity of data on tDCS’ effect on cognitive function during Ramadan fasting. This paper aims to assess the effect of tDCS of three brain areas, including the right dorsolateral prefrontal cortex (DLPFC), posterior parietal cortex (PPC), and cerebellum on cognitive function, and obtain safety data in healthy adults during Ramadan fasting.

**Methods and material:**

A total of 42 healthy, right-handed participants were randomly assigned to one of the 6 stimulation groups: active (anodal)-tDCS of right DLPFC, PPC, and cerebellum; or sham for DLPFC, PPC, and cerebellum after 8 h of fasting for Ramadan. Safety data and cognitive function, such as attention-switching tasks (AST), were obtained by employing the Cambridge Neuropsychological Test Automated Battery (CANTAB) before and after each tDCS session. The cognitive function outcome variables were the response time and the percentage of correct answers in AST. For sham stimulation, the placement of the electrodes was the same as for the active stimulation.

**Results:**

An improvement in performance time in attention tasks was observed; however, it did not reach a significant level after anodal stimulation of the DLPFC, PPC, and cerebellum. Overall, there were no statistically significant differences between the active and sham tDCS groups in cognitive function. There were no significant side effects of tDCS during fasting for any group.

**Conclusions:**

Our data suggest that there are variable effects of tDCS on attention tasks during Ramadan fasting. TDCS appears to be safe, well-tolerated and adhered to the international standard of safety in the local population during Ramadan fasting. Further large sample size studies should be conducted to validate the current study findings and reach better conclusions.

## Highlights

• There is a paucity of data about Transcranial direct current stimulation (tDCS) effect on cognitive function during Ramadan fasting.
• tDCS appeared to be safe, well-tolerated and adhered to the international standard of safety during Ramadan fasting.
• This creates an exciting opportunity to develop this approach as a therapeutic intervention.

## Introduction

Transcranial direct current stimulation (tDCS) is one of the non-invasive brain stimulation methods that is increasingly used in basic neuroscience research (Nitsche and Paulus, 2001; Stagg and Nitsche, 2011; Cappon et al., 2016), or to evaluate the possible therapeutic effects in neurological and psychiatric disorders (Woods et al., 2016; Kuo, and Nitsche, 2012; Flöel, 2014; Kuo et al., 2014; Bennabi and Haffen, 2018). One of the commonly used methods delivers tDCS at an intensity of 1–2 mA (0.029–0.057 mA/cm^2^) through pad electrodes that are placed on the scalp with a current that flows from the anodal (depolarizing cortical neurons and increases neural activity) to cathodal (hyperpolarizing neurons and reduces neural activity) electrode (Nitsche and Paulus, 2001; Stagg and Nitsche, 2011). tDCS has significantly developed in the last few years with more than 1500 research articles recently published on the topic (Flöel, 2014; Kuo et al., 2014; Rabipour et al., 2019; Bikson et al., 2016). tDCS modulates a variety of psychological processes such as motor functions and cognitive control; however, the reported data in the literature has showed conflicting reports about increasing and decreasing cognitive performance (Jacobson et al., 2012; Horvath et al., 2015; Dwyer et al., 2019).

Fasting during the month of Ramadan is a religious practice for Muslims all over the world. There are about 2 billion Muslims, and hundreds of millions fast every year. Fasting affects the circadian rhythms and the biorhythms of nutrient consumption, which results in changes in physiological, cognitive, behavioral, and metabolic functions, as well as sleep patterns (Roky et al., 2004; Bahijri et al., 2013; Ibrahim et al., 2008; Yucel et al., 2004; Norouzy et al., 2013; Salahuddin et al., 2014). Moreover, studies have shown the effects of fasting on visual learning and working memory (Tian et al., 2011; Ajabnoor et al., 2014; Ho-Heng Tian, 2018).

With the advent of modern computerized cognitive testing batteries such as Cambridge Neuropsychological Test Automated Battery (CANTAB), it has become possible to allow for hypothesis-driven exploration of different domains of cognition testing with certainty (Faisal et al., 2019; Habib et al., 2018; Al-Thaqib et al., 2018; Bashir et al., 2017). Its application has an advantage, as it is a computerized test and takes less time compared to traditional pen-and-paper cognitive assessment tasks. Moreover, it gives more accurate results, particularly in tasks requiring counting time and response delay, such as attention-switching tasks (AST). We conducted this study to examine the effects of tDCS in healthy fasting individuals for cognitive function, particularly in the attention networks. The “attention system” is a top-down process, consciously coordinating and reorganizing new information. It is known as working memory (Shallice, 1988). Anatomically, working memory is located within the neural circuits connecting the dorsolateral, ventrolateral and orbitofrontal structures (Shallice, 1988; Gazzaley and Nobre, 2012; Zeamer and Fox Tree, 2013; Mishra et al., 2013; Zanto et al., 2011; Freund, 2003). Executive functions including planning ability, response inhibition, and working memory. They are essential tools for an adjustment to daily life activities required for cognitive flexibility and control of our emotions and behavior so we can successfully act in a goal-directed manner. Cognitive control over attention is particularly important to simply focus on task-relevant information and to not be distracted by irrelevant stimuli (Ochsner and Gross, 2005).

The frontal lobes account for two-thirds of the human brain. Frontal lobe functions include motor functions and cognition processes, such as executive function, attention, memory, and language (Grimaldi et al., 2017; Ferrucci et al., 2017). In addition, it constitutes affect, mood, personality, and self-awareness, as well as social and moral reasoning (Garon et al., 2017).

The posterior parietal cortex (PPC) plays a critical role in attentional processing. Top-down attentional control relies on the superior part of the PPC, which includes the intraparietal sulcus (Corbetta et al., 2008). For example, covert attention toward an instructed spatial location produced sustained activation of the intraparietal sulcus (Corbetta et al., 2008). On the other hand, stimulus-driven attention reorientation depends on the temporal-parietal junction (TPJ), which consists of the inferior part of the PPC and the superior part of the temporal cortex (Corbetta et al., 2008).

The cerebellum has a distinguished role in controlling both motor and cognitive functions. In 1998, Schmahmann and Sherman postulated the existence of a “cerebellar cognitive-affective syndrome”, which has been attributed to the disruption of the neural circuits linking prefrontal, temporal, posterior parietal and limbic cortices with the cerebellum (Schmahmann and Sherman, 1998; Miquel et al., 2019). Since prefrontal and posterior parietal neural circuits are considered crucial for attention, the close anatomical connections to the cerebellum indicate a cerebellar relevance for these functions as well. There has also been evidence of neurofunctional activation of the cerebellum during attention tasks (O’Halloran et al., 2012).

We hypothesized that a tDCS session performed over the right dorsolateral prefrontal cortex (DLPFC), posterior parietal cortex (PPC), and cerebellum in healthy subjects after 8 h of fasting can influence and improved their cognitive function. There is no sign for irreversible brain damage produced by tDCS protocols within a wide range of stimulation parameters (≤ 40 min, ≤ 4 mA, ≤ 7.2 C) (Bikson et al., 2016). The second objective of the study was to investigate the safety and tolerability aspects of 1.5 mA tDCS over the three brain areas during Ramadan fasting.

## Material and methods

This study was a parallel randomized single-blind sham-controlled study where each participant took part in one of the six stimulation conditions.

### Participants

Forty-two healthy participants (21 men aged between 18 and 30 years with mean ± SD; 22.9 ± 3.3 were randomly assigned to one of the six stimulation groups: (1) anodal tDCS of right DLPFC; (2) anodal tDCS of right PPC; (3) anodal tDCS of right cerebellum; (4) sham tDCS of right DLPFC; (5) sham tDCS of right PPC; and (6) sham tDCS of the right cerebellum. All participants were right-handed according to the Edinburgh Handedness Inventory (Oldfield, 1971). Exclusion criteria for participation in the experiments were: (1) having contraindications for receiving tDCS, e.g., a history of seizures or hereditary conditions, having any metal in their head, severe headaches, or pregnancy; (2) current usage of any medicine that could affect brain excitability, motor learning or cognition; (3) a history of neurological or psychiatric diseases; (4) disability in finger(s), hand(s) or wrist(s), (5) age above 40 years or less than 18 years. All groups were matched for age, ethnicity, gender, and socioeconomic status. This study was conducted during the month of Ramadan at the Department of Physiology, College of Medicine and King Khalid University Hospital (KKUH). The IRB committee from KKUH approved the project. Informed consent was obtained from each subject. This study was conducted in accordance with the Declaration of Helsinki. All tests were conducted between 11AM and 4PM. All procedures required around 1 h to complete.

### Procedures and materials

**Fig. 1:**
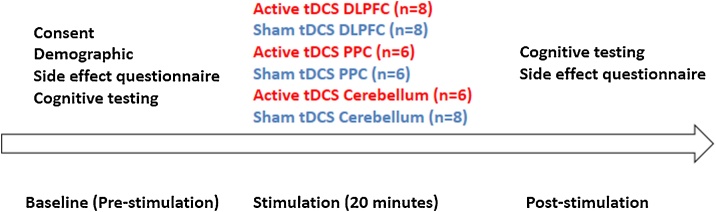
Work flow of experimental design and area of stimulation before and after anodal active and sham stimulation for right dorsolateral prefrontal cortex (DLPFC), posterior partial cortex (PPC) and cerebellum.

As shown in Fig. 1, the demographic and safety of tDCS questionnaires were completed in the screening section. Participants then performed a cognitive function test using the CANTAB research suite software (version 6. 0.37, Cambridge Cognition, Cambridge, UK). Stimulation was delivered through a constant current with 1.5 mA current for 20 min either active or sham stimulation generated by a (Soterix Medical Inc., NY) with two 35 cm^2^ (5 cm × 7 cm) electrodes on saline-soaked sponges. The active electrode was placed on either the right DLPFC, PPC or the cerebellum and reference electrode on the contralateral side. A sham (control) stimulation was performed in which the electrodes were placed on the same location as active anodal stimulation but the current was ramped up to 1.5 mA over 30 s and then back down at the beginning and end of 20 min. Following the completion of the stimulation, participants completed the cognitive and safety assessments again (Fig. 1).

### Assessment of adverse events assessment

Each subject was given an adverse event questionnaire that had been translated into Arabic after each one of the stimulation sessions that inquired about the presence or absence of the following symptoms: tingling, itching sensation, burning sensation, neck pain, scalp pain, headache, fatigue, difficulties in concentration, nervousness, sudden mood change, change in visual perception, unpleasant sensation, visual sensation, nausea, drowsiness, and whether the subject still feels the stimulation or not (Faisal et al., 2019). Each one of the previous potential adverse events was rated from 1 to 5 (1 = very mild; 5 = very severe).

### Cognitive function

#### Attention switching task (AST)

AST is used for the sensitive measure of top-down cognitive control processes as an executive function by the participant’s ability to switch attention between the direction of an arrow, which would appear on the right or left side of the screen, and could point in left or right directions and its location on the screen (Faisal et al., 2019; Habib et al., 2018 Al-Thaqib et al., 2018 Bashir et al., 2017). Each trial displayed a cue at the top of the screen that indicates to the participants whether they should press the right or left button according to the “side on which the arrow appeared” or the “direction in which the arrow was pointing”. The parallel of AST was used for all experiments to roll out of possible learning effect.

### tDCS

A commercially available stimulator (Soterix Medical Inc., NY) was used to deliver direct current with an intensity of 1.5 mA for 20 min through a pair of saline-soaked rectangular sponge surface electrodes. The size of active and return electrodes were 35 cm^2^ (5 cm × 7 cm), respectively. In this study, we used the current intensity in a safe range (Nitsche and Paulus, 2001; Poreisz et al., 2007), to modulate the excitability of neurons in the target area (Bastani and Jaberzadeh, 2013a,b; Vaseghi et al., 2015a,b; Faisal et al., 2019). Therefore, the active electrode was placed over the target areas (right DLPFC, PPC, or cerebellum) and the return electrode was fixed over the contralateral supraorbital region. For the sham group, the active electrode was placed on the same position (right DLPFC, PPC or cerebellum). The distribution for the stimulation conditions was randomly balanced across participants. The current was ramped up to 1.5 mA and then ramped down so that participants felt an initial sensation for 30 s of stimulation.

The locations of right DLPFC, PPC or cerebellum were determined using the international 10–20 system (Steinmetz et al., 1989). Therefore, the stimulating electrodes for DLPFC or PPC were placed over F4 and P4, respectively. For the cerebellum tDCS stimulation, the active electrode was placed over the right cerebellar cortex (3 cm lateral to the inion), and the reference electrode was positioned on the skin area overlying the right buccinator muscle. Participants were asked to report tDCS side effects such as itching, tingling, burning sensations, headache, pain, and any other sensations (Faisal et al., 2019).

### Statistical analysis

Data were analyzed using SPSS (IBM Corp. Released in 2012. IBM SPSS Statistics for Windows, Version 21.0. Armonk, NY: IBM Corp.).

The AST has the following outcome measures in response time (AST congruency cost (mean, correct), AST switching cost (mean, correct), AST mean correct latency, AST mean correct latency (congruent), AST mean correct latency (incongruent), AST mean correct latency (blocks 3, 5) (no switching blocks), AST mean correct latency (block 7) (switching block) and AST percent correct trials.

Pre-stimulation formed the baseline measurement for all subsequent measures. To measure the acute effects of stimulation on cognitive function for each site of stimulation (DLPFC, PPC, and cerebellum) with 2 × 2 mixed ANOVAs [Time (pre/post-stimulation) × Condition (sham/active)] were performed on performance time measures of AST, with time as within-subjects and conditions as between-subjects factors. Follow-up *t*-tests were then used to investigate the effects of the within- and between-subjects factors.

The safety data were qualitative and the assumption of expected frequency being <20% was not violated for tingling, itching, burning, headache, or feeling the stimulation on the right side after removing the electrodes. We used Pearson’s chi-square test for comparing the presence of these side effects before and after stimulation. As the expected frequency assumption was violated for fatigue, difficulty concentrating, acute mode change, change in visual perception, unpleasant sensation, unpleasant sensation in vision, nausea, drowsiness, and feeling the stimulation on the right side after taking off the electrodes, we used Fisher’s exact test for these side effects. Statistical significance was set at p < 0.05.

## Results

Participants were randomly assigned to the brain area of stimulation (DLPFC, PPC, and cerebellum) and order of stimulation (active anodal or sham). The six resulting groups showed no significant differences with respect to age, gender, education, or body mass index. Table 1 shows the demographic data.

**Table 1:** Demographic Characteristics.

Variable	DLPFC		PPC		Cerbellum	
	Active	Sham	Active	Sham	Active	Sham
Number (Female/Male)	5/3	4/4	3/3	3/3	3/3	3/5
Age (years)	22.5 ± 2.6	23.8 ± 1.4	22.7 ± 4.8	22.5 ± 1.8	21.6 ± 3.8	23.5 ± 2.6
BMI (kg/m^2^)	26.1 ± 3.6	26.7 ± 2.4	25.7 ± 2.9	26.8 ± 4.2	26.3 ± 2.7	25.9 ± 4.3

Response time (RT) measured for (AST congruency cost, AST switching cost, AST correct latency, AST correct latency (congruent), AST correct latency (incongruent), AST correct latency (blocks 3, 5) (no switching blocks), AST correct latency (block 7) (switching block) and AST percent correct trials (mean ± SD) are reported in Table 2, Table 3, Table 4 for all four groups at each experimental session. The results of ANOVA showed no significant differences in RT or percent correct trials (p < 0.05) at baseline among the groups for six experimental sessions.

**Table 2:** Cognitive function through attention switching task (AST) for active and sham groups for right dorsolateral prefrontal cortex.

Variable	Active			Sham		
	Pre ± SD	Post ± SD	p-value	Pre ± SD	Post ± SD	p-value
AST Congruency cost (Mean, correct)	81.9 ± 70.2	82.1 ± 63.9	0.99	83.0 ± 32.7	84.3 ± 76.0	0.96
AST Switching cost (Mean, correct)	209 ± 187	153 ± 116	0.48	199 ± 142	180.5 ± 66.1	0.73
AST Mean correct latency	683 ± 155	642 ± 154	0.69	677 ± 190	612 ± 133	0.44
AST Mean correct latency (congruent)	656 ± 135	604 ± 132	0.08	647 ± 182	626 ± 101	0.52
AST Mean correct latency (incongruent)	708 ± 188	646 ± 180	0.24	711 ± 200	670 ± 165	0.67
AST Mean correct latency (blocks 3, 5) [non-switching blocks]	593 ± 104	589 ± 104	0.95	604 ± 197	579 ± 130	0.32
AST Mean correct latency (block 7) [switching block]	723 ± 240	683 ± 216	0.61	753 ± 209	699 ± 146	0.42
AST Percent correct trials	93.1 ± 4.34	93.2 ± 4.4	0.95	91.1 ± 7.4	90.5 ± 5.4	0.58

**Table 3:** Cognitive function through attention switching task (AST) for active and sham groups for posterior parietal cortex (PPC).

	Active			Sham		
Variable	Pre ± SD	Post ± SD	P-value	Pre ± SD	Post ± SD	P-value
AST Congruency cost (Mean, correct)	77.2 ± 70.0	112.8 ± 82.4	0.51	84.9 ± 56.2	61.9 ± 32	0.32
AST Switching cost (Mean, correct)	217.2 ± 151	252.8 ± 118	0.54	224.0 ± 117	236.6 ± 60	0.62
AST Mean correct latency	619 ± 113	599 ± 108	0.62	623 ± 157	595 ± 155	0.52
AST Mean correct latency (congruent)	602 ± 103	581 ± 96	0.67	604 ± 156	616 ± 143	0.62
AST Mean correct latency (incongruent)	703 ± 134	658 ± 143	0.08	708 ± 166	697 ± 168	0.84
AST Mean correct latency (blocks 3, 5) [non-switching blocks]	568 ± 66	549 ± 100	0.43	603 ± 152	598 ± 150	0.82
AST Mean correct latency (block 7) [switching block]	748 ± 187	731 ± 156	0.37	740 ± 179	734 ± 166	0.82
AST Percent correct trials	95.4 ± 2.7	94.1 ± 2.2	0.43	88.6 ± 9.7	95.8 ± 2.5	0.16

**Table 4:** Cognitive function through attention switching task (AST) for active and sham groups for cerebellum.

	Active			Sham		
Variable	Pre ± SD	Post ± SD	P-value	Pre ± SD	Post ± SD	P-value
AST Congruency cost (Mean, correct)	78.3 ± 68.13	80.6 ± 16.4	0.62	77.4 ± 55.6	76.2 ± 23.0	0.86
AST Switching cost (Mean, correct)	203.2 ± 97	199 ± 101	0.40	216 ± 111	204 ± 127	0.63
AST Mean correct latency	690 ± 161	637 ± 169	0.62	637 ± 113	634 ± 93	0.47
AST Mean correct latency (congruent)	652 ± 134	604 ± 173	0.13	632 ± 117	615 ± 93	0.19
AST Mean correct latency (incongruent)	730 ± 194	673 ± 165	0.07	747 ± 115	705 ± 95	0.21
AST Mean correct latency (blocks 3, 5) [non-switching blocks]	564 ± 130	529.0 ± 135	0.68	596 ± 110	588 ± 85	0.18
AST Mean correct latency (block 7) [switching block]	737 ± 213	688 ± 220	0.09	748 ± 152	686 ± 138	0.08
AST Percent correct trials	92.1 ± 4.5	93.9 ± 3.6	0.51	93.8 ± 4.0	94.9 ± 5.4	0.67

**Fig. 2:**
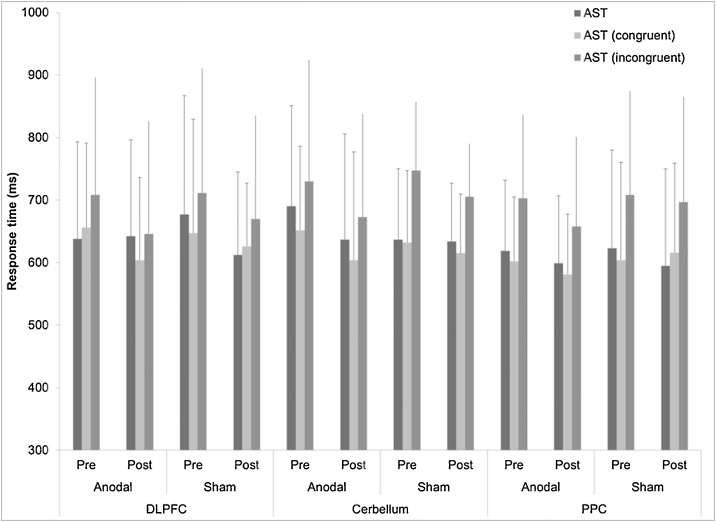
Comparison of attention switching task for mean correct latency, congruent and incongruent condition before and after anodal and sham stimulation for right dorsolateral prefrontal cortex, posterior partial cortex and cerebellum. Error bars are standard deviation.

Fig. 2 shows the mean RT (ms) for AST correct latency, AST correct latency (congruent), and AST correct latency (incongruent) before and after interventions (active/sham) over two-time points (baseline/post) in all six groups.

### Dorsolateral prefrontal cortex (DLPFC)

A no main effect of Time and Condition was observed for AST Mean correct latency [F = 4.742, p = 0.11 for time and F = 2.678, p = 0.38, for condition], AST Mean correct latency (congruent) [F = 3.246, p = .24 for time and F = 3.222, p = 0.18, for condition] and AST Mean correct latency (incongruent) [F = 3.920, p = 0.20 for time and F = 3.674, p = 0.32, for condition].

In addition, there was a decrease in scores from pre- to post-stimulation, but did not reach significant (p = > 0.05, Fig. 1, Table 2) difference for the active condition and for the sham condition. See Table 2 for value for AST variables for the active and sham groups.

The most frequent side effects was reported in the active group but did not reach significance (headache p = 0.186, unpleasant sensation p = 0.122). However, fewer adverse effects were reported in the sham group (headache p = 0.144, unpleasant p = 0.271).

### Posterior parietal cortex (PPC)

A no main effect of Time and Condition was observed for AST Mean correct latency [F = 4.102, p = 0.31 for time and F = 4.022, p = 0.24, for condition], AST Mean correct latency (congruent) [F = 4.102, p = 0.38 for time and F = 4.322, p = 0.42, for condition] and AST Mean correct latency (incongruent) [F = 3.820, p = 0.62 for time and F = 4.230, p = 0.41, for condition]. However, we did observe a significant Time × Condition interaction for incongruent condition only [F = 6.244, p = 0.05].

In addition, we did observe a decrease in scores from pre- to post-stimulation, but it did not reach a significant (p = >0.05, Fig. 1) difference for the active and sham conditions. Except for improvement of the active condition only for incongruent condition (p = 0.08).

See Table 3 for value for AST variables for the active and sham groups. There was no significant effect observed in both conditions (p > 0.05).

The side effects were reported in the active group but did not reach significance (tingling p = 0.07, sudden mood change, p = 0.09), as the participants in the sham group showed a similar side to active group (tingling p = 0.07, sudden mood change, p = 0.22).

### Cerebellum

A no main effect of Time and Condition was observed for AST Mean correct latency [F = 4.228, p = 0.26 for time and F = 3.878, p = 0.20, for condition], AST Mean correct latency (congruent) [F = 4.210, p = 0.40 for time and F = 4.180, p = 0.48, for condition] and AST Mean correct latency (incongruent) [F = 4.920, p = 0.32 for time and F = 4.476, p = 0.36, for condition]. However, we did observe a significant Time × Condition interaction for incongruent condition only [F = 6.170, p = 0.04].

There was an improvement in the active condition only for incongruent condition (p = 0.07).

See Table 4 for value for AST variables for the active and sham groups. There was no significant effect observed in either condition (p > 0.05).

The most commonly reported side effects in the active group were (burning p = 0.05, difficulty in concentration p = 0.44, sudden mood change p = 0.08, unpleasant sensation in vision p = 0.22) after anodal cerebellum stimulation. However, there were fewer adverse effects reported in the sham group as (burning p = 0.15, difficulty in concentration p = 0.42, sudden mood change p = 0.24, unpleasant sensation in vision p = 0.42).

## Discussion

This study investigated cognitive function for attention and the safety aspect of tDCS in a cohort of healthy individuals practicing Ramadan fasting. Focusing on the attention domain, we investigated how tDCS over the DLPFC, PPC, and cerebellum affects attentional processing in healthy subjects during Ramadan fasting. There was an improvement in performance time assessed by AST but it did not reach the significance level after tDCS stimulation. There were no serious side effects from stimulation during fasting.

There are mixed reports on the effects of Ramadan fasting on cognitive parameters (Bahijri et al., 2013; Ibrahim et al., 2008; Norouzy et al., 2013 Salahuddin et al., 2014, Tian et al., 2011; Ho-Heng Tian, 2018). A fair number of studies have investigated the effects of tDCS on these three areas’ function in healthy and ill subjects without fasting (Woods et al., 2016; Flöel, 2014; Bennabi and Haffen, 2018; Carlos et al., 2017; Manuel, Schnider, 2016; Klaartje et al., 2016). One study demonstrated that right frontal anodal transcranial direct current stimulation resulted in a possible increase in task control of healthy right-handed individuals (Avenanti et al., 2017). Another study examined the effect of anodal tDCS of the right PFPC on visual working memory and found that it facilitated attention control and improved attention scope (Westwood et al., 2017). In addition, Anodal tDCS stimulation of the frontal lobe had no effect on picture naming tasks, according to one study set to examine the effects of tDCS on language (Gutierrez et al., 2001). Although we expected single-session focal stimulation a-tDCS over DLPFC, PPC or cerebellum led to enhance performance time, compared to the sham group, due to neuropsychological evidence strongly supports the role of PPC or DLPFC in higher cognitive functions or sensorimotor integration (Bahrick et al., 1954; Seger, 1994; Castro-Alamancos et al., 1995; Castro-Alamancos and Connors, 1996), no specific effects were found on AST.

The absence of any effects for DLPFC or PPC tDCS in the current study can be explained by tDCS characteristics or task-dependent effects of tDCS on learning and memory formation (Saucedo Marquez et al., 2013). The standard tDCS montage (the current intensity (1–2 mA) and electrode size (25–35 cm2) on different areas of the brain could positively affect the motor learning task (Ammann et al., 2016). In spite of that, previous study using the standard intensity and electrode size not to improve sensorimotor learning with single session application in healthy participants (Hashemirad et al., 2016, 2017; Convento et al., 2014).

Another a possible reason can explain inter variability between participants (Lopez-Alonso et al., 2014) for the not improving of the cognitive task in the current study (Hashemirad et al., 2017). Further research is needed to compare the effects of different protocols of tDCS in terms of intensity, electrode size as well as stimulation sites on cognitive tasks during fasting.

Safety and toxicity are additional important major concerns with regard to online tDCS that must be addressed for healthy subjects in fasting, although tDCS differs in many aspects from other non-invasive tES therapies for weak electric currents that do not induce directly neuronal action potentials (Bikson et al., 2016). It has been used worldwide in thousands of subjects with no reports of any toxic effects until now (Bikson et al., 2016). Therefore, addressing tDCS dosage parameters: current dosage (measured in amperes); duration of stimulation; and electrode montage (size and position of all electrodes), is critical for a safe application of tDCS during fasting. The side effects most commonly reported are mild headache, tingling, itching, burning sensation, and skin redness under the area of electrodes (Bikson et al., 2016). Our results are in line with these findings. However, we also found a low frequency of these side effects. In our study, we did not find a significant difference in the number of side effects reported between the active and sham stimulation groups for any of the interventions.

### Limitations

There are some limitations to this study. One limitation of the present study was its small sample size for each brain area, which may have been inadequate to detect statistical differences for some parameters. Fasting in Ramadan is observed only for one month, which limited our ability to collect more samples. We recommend that future studies increase the sample size. We included healthy young individual participants, thus we could not extrapolate our results to elderly or patient’ populations. Regarding the lack of effects of a-tDCS on response time in AST, one possible reason for the null findings may be related to the size of the stimulating electrodes. Further research is recommended over the brain areas using different electrode sizes.

We assessed outcome measures only immediately after the intervention, and long term effects of tDCS on behavioral outcome measures were not demonstrated in this study.

Furthermore, the present study could have been improved by using two groups with measurements made before, during, and after the fasting period. Ramadan fasting involves total abstinence from not only food but also fluids, which could affect the brain process of cognition. In the current study, we did not examine the effect of dehydration using a direct measure of body water content such as serum osmolality.

### Conclusion

Our results demonstrated that a single session a-tDCS over DLPFC, PPC or cerebellum for AST had no significant additional effects on response time in a fasting condition. tDCS was found to be safe, well-tolerated and adhered to the international standard of safety in the local population during Ramadan fasting. Furthermore, more studies with larger sample sizes should be conducted to validate the current study findings.

## Financial support

None.

## Conflicts of interest

None.

## CRediT authorship contribution statement

**Fahad Alsultan:** Methodology, Investigation, Data curation, Writing - original draft, Writing - review & editing. **Malak Alaboudi:** Methodology, Investigation, Data curation, Writing - original draft, Writing - review & editing. **Abdullah Almousa:** Conceptualization, Methodology, Formal analysis, Investigation, Data curation, Writing - original draft, Writing - review & editing. **Reema Alajaji:** Data curation, Formal analysis, Methodology, Writing - original draft, Writing - review & editing. **Shahid Bashir:** Conceptualization, Formal analysis, Investigation, Writing - original draft, Writing - review & editing, Supervision.
